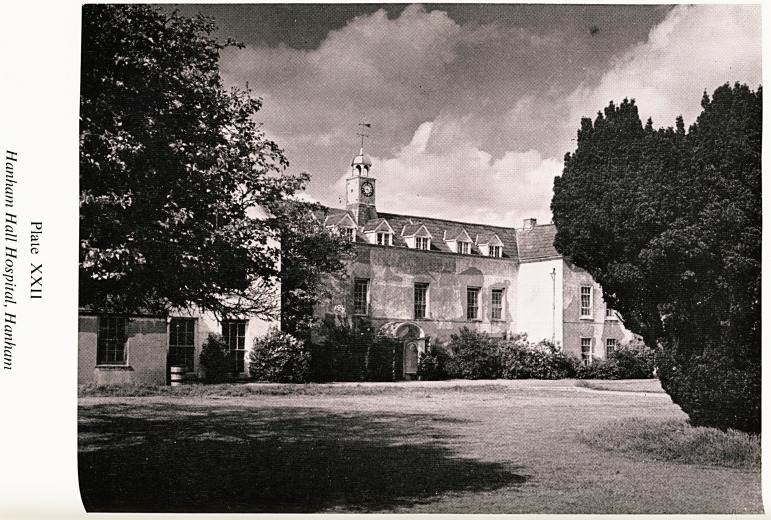# Sixty Years of Stoke Park Hospital

**Published:** 1969-07

**Authors:** J. Jancar

**Affiliations:** Consultant Psychiatrist—Stoke Park Hospital Group, Bristol


### SIXTY YEARS OF STOKE PARK HOSPITAL
(1909—1969)

by

J. JANCAR, M.B., B.Ch., B.A.O., D.P.M.

*Consultant Psychiatrist — Stoke Park Hospital Group, Bristol*

---

*"The further backward you can look,*
*the further forward you are likely to see"*
*—Sir Winston Churchill.*

### INTRODUCTION

Within the grounds of Stoke Park Hospital is a memorial clock tower with the following inscription:—

In memory of

HAROLD NELSON BURDEN, Priest of Clevedon Hall, Somerset.

1859—1930

He gave this estate in trust for the nation. A man of vision, faith, genius and unfailing courage. A pioneer in mental work and research. He lived arduous days and had the joy of seeing the fruition of his hope and lifelong efforts.
Thanks be to God.

This inscription epitomises well the life, work and personality of the founder of Stoke Park Hospital.

As a young man, Mr. Burden appeared to be determined to devote his life to the welfare of others, when deciding, against the wishes of his family, to enter the Church. After his ordination at Carlisle in 1888, he spent some time in curacies in the East End of London. There he first came in contact with the people in real need and in real misery. These early experiences undoubtedly influenced and shaped much of Mr. Burden's later life and work. Soon after, Mr. and Mrs. Burden went to Canada as missionaries to work amongst the Ojibway Indians and lumbermen. In his book "Life in Algoma", published 1894, Mr. Burden portrayed their life and early struggles in the lone lands. The death of their two children and Mr. Burden's poor health compelled them to leave Canada in 1891. On his return to England he was a curate in Shoreditch and later at Milton, Cambridgeshire, until 1898, when he was appointed the chaplain of Horfield Prison in Bristol.

His wife visited the homes of prisoners and was impressed by the things she saw — the effect of a bad home on the mentally retarded child, who at that time could not attend a day school, and the excessive indulgence in alcohol, especially in women who were in and out of the Police Courts because of drunkenness. The latter led to Mr. and Mrs. Burden's becoming instrumental in the founding, in 1889, of Brentry Institution for Inebriates in Bristol. In 1921, Brentry became an Institution for the mentally retarded, and after 1948 merged with Hortham Colony (which was built and opened in 1932, by the Bristol City Authorities) forming the Hortham/Brentry Hospital Group. Soon after the opening of Brentry Institution, Mr. and Mrs. Burden purchased and opened a small home for mentally retarded children in Horfield, Bristol, named the Royal Victoria Home.

In 1902, Mr. Burden founded the Incorporation known as "National Institutions for Persons Requiring Care and Control" and became the first Warden. Methodical and businesslike in his management, Mr. Burden had a brain for finance, and was consequently able to administer the huge undertaking into which his modest beginnings grew with efficiency, common sense, and a sound economy.

In 1904, Mr. Burden was appointed by the Government to be a member of the Royal Commission charged with the inquiry into the care of the "feebleminded". The result of the Royal Commission was the Mental Deficiency Act of 1913, under which "Stoke Park Colony" was the first in the British Isles to be certified as an institution for mentally retarded patients. This inquiry and the Commission's visits to the Continent inspired Mr. Burden to devote the rest of his life and financial resources to the care and welfare of mentally retarded people.

Mr. and Mrs. Burden first bought Eastern Counties Institution, East Harling, Norfolk, in 1904 and then Sandwell Hall, Handsworth, Staffordshire, in 1906. In 1909 they acquired from the Duke of Beaufort the Dower House, Stoke Park, which became the nucleus of the group of institutions later known as Stoke Park Colony. Whittington Hall, Chesterfield, was opened in 1912.

### Early Years

Stoke Park Colony was opened on the 1st of April, 1909. The staff consisted of a controller, four matrons, three certified teachers, and nurses and attendants, making a total of 48. A medical officer visited daily. In 1914, Mr. and Mrs. Burden moved to Clevedon Hall in Somerset (now St. Brandon's School), where children from Stoke Park went for holidays and special domestic training was given to some high-grade mentally defective girls. The founders were very concerned with rehabilitation of the patients and believed that this could be done by teaching the children and young patients some useful occupation. Accordingly, they provided training schemes for laundry, housework, weaving, gardening, carpentry, boot-making, tailoring, brush-making, market gardening and farm work. They were also interested in the physical treatment of patients. Heliotherapy and open-air treatment were known at that time to increase vitality, weight, and resistance to disease; and five two bedded revolving houses were built among the trees near the hospital ward for convalescent patients, and a sheltered enclosure between the wards was used for sun baths. During the summer large tents were provided as dormitories, dining rooms and play rooms.

As the demand for more beds grew, Mr. Burden purchased more property, and Heath House was acquired in 1911 and Stapleton Grove-Beech House, West Side (now Purdown Hospital) in 1916, followed by Hanham Hall and Leigh Court in 1917. Additional wards were later built at Stoke Park and West Side.

Mr. and Mrs. Burden were also concerned with the religious life of the patients and staff and they provided chapels in all the hospitals. Chaplains were appointed who regularly visited the patients at their work in the wards. These religious facilities continue today.

Mrs. K. M. Burden died in 1919, and Mr. Burden married Miss R. Williams, who was the Superintendent of Stoke Park.

*Research at Stoke Park*

Mr. Burden was not content merely to offer custodial care for mentally retarded patients but made financial provision for and encouraged research into the causes, treatment and prevention of mental retardation.

In 1927, he appointed Dr. R. W. Braithwaite as Director of Medical Services. Splendid laboratories, which included X-ray plant and clinical photography, were built and liberally equipped to enable research work to commence on the scale planned by Mr. Burden. Dr. Braithwaite died in 1929, and in 1930 Professor R. J. A. Berry was appointed Director of Medical Services.

Mr. Burden (Plate XIV) died on 15th May, 1930. Just before his death he approved a scheme for the further development of research work at Stoke Park and ancillary hospitals, which by then had over 2,000 beds, and increased the financial promotion far beyond the more modest scale first suggested.

The research team consisted of the following members:—

| | |
|---|---|
| Professor R. J. A. Berry | Director of Medical Services. (Plate XV) |
| Dr. R. M. Bates | Resident Medical Officer (Plate XVI) |
| Dr. R. M. Norman | Assistant Medical Officer. |
| Professor J. A. Nixon | University of Bristol; Consulting Physician. |
| Professor E. W. Hey Groves | University of Bristol; Consulting Surgeon. |
| Professor I. Walker Hall | University of Bristol; Consulting Pathologist and Bacteriologist. |
| Dr. R. G. Gordon | Consulting Neurologist and Psychologist. |
| Mr. A. E. Iles | Bristol General Hospital; Consulting Ophthalmologist. |
| Mr. J. Angel James | Bristol Royal Infirmary; Consulting Laryngologist. |
| Dr. T. B. Wansbrough | Consulting Radiologist. |
| Dr. Evelyn R. Bates | Clinical Pathologist. |
| Mr. W. J. Jones | Dental Surgeon. |

In addition to these, there were six psychological and technical staff employed to help with the research. (One of them, Miss D. Sperrin West is still working at Stoke Park as part-time psychologist).

Professor Berry established a teaching museum of pathological specimens of the brain and other tissues. Stoke Park also became recognised by the Universities of London and Bristol as a teaching institution for candidates for D.P.M. (Mental Deficiency).

The results of the research were published in Stoke Park Studies — First Series (Berry, 1933).

The importance of well-trained nursing staff as part of the hospital team was always recognised at Stoke Park. Originally nurses were given lectures by medical and other senior staff only. In 1933, the first sister tutor was appointed, and in 1935 the Nurses' Training School was opened. The same year it was recognised by the Royal Medico-Psychological Association as a training school, and in 1941 received the same recognition by the General Nursing Council of England and Wales.

New school buildings for the patients were opened at Stoke Park in 1933, giving greater facilities to the teachers to continue their successful programmes and advance to present-day methods of teaching at Stoke Park and Purdown Hospitals.

### Later Developments

In 1933, Mrs. Burden donated the sum of £10,000 and with the gift expressed her desire that it should primarily be devoted to problems underlying the causation and inheritance of normal and abnormal mentality. The Burden Mental Research Trust came into being.

*The Burden Mental Research Trust* was administered by a Committee of Management consisting of the following bodies officially represented:—

The British Medical Association.
The Medical Research Council.
The Board of Control.
The Board of Education.
The Galton Eugenics Laboratory of the University of London.
The Royal Medico-Psychological Association.
The Central Association for Mental Welfare.

The Committee of Administration made the following major appointments:—

The Director and Principal Investigator: Dr. J. A. Fraser Roberts. (Plate XVII).
Part-time Assistant: Dr. R. M. Norman. (Plate XVIII).
Psychological Assistant: Dr. Ruth Griffiths.

They also created, apart from some other minor appointments, the important post of part-time social worker.

Research has been carried out along three main lines with the common purpose of enquiring into the causation of mental abnormalities and comparison with the mentally normal:—

Plate XIV

*The Rev. Harold Nelson Burden, M.A., Founder of Stoke Park Hospital.*

Plate XV

*Prof. R. J. A. Berry, M.D., F.R.C.S. Ed., F.R.S. Ed.*

Plate XVI

*Dr. R. M. Bates, O.B.E., F.R.C.S., D.P.M.*

Plate XVII

*Dr. J. A. Fraser Roberts, C.B.E., M.A., F.R.C.P., F.R.S.*

Plate XVIII

*Dr. R. M. Norman, M.D., F.R.C.P., D.P.M.*

1. A complete ascertainment of the mental functions of cross-sections of a mentally normal school population. 3,400 children from Bath were mentally tested, whose homes were within the City boundaries and who were born between 1st September, 1921 and 31st August, 1925, inclusive. Later, a psychological assessment of 2,000 normal children from a Bristol school was undertaken.

2. An analogous study of a known mentally retarded population—the investigators mentally tested every new admission to Stoke Park Colony, and by 1937 had over 1,000 case records. Mental testing of every admission to Stoke Park has continued to the present date.

3. An examination after death of the brains of individuals drawn from the population mentioned, with a view to ascertaining how and why the brains of the mentally abnormal differ from those of normal mentality. Up to October 1937, 123 brains of defectives and 82 from persons of normal mentality had been macro- and microscopically examined. Professor S. E. Whitnall of the University of Bristol later joined the team, carrying out an investigation on the calcarine and visual areas of the brain. Many observations were made, and abnormalities of the brain noted, and reported by the investigators, including a congenital form of amaurotic familial idiocy, which is known in the literature as Norman's disease or Norman-Wood disease (Norman and Wood, 1941).

When, in 1936, a Child Guidance Clinic was opened in Bristol, Dr. Griffiths assisted with the work there and so forged the link between community care and hospital research.

The results of the Burden Mental Research Trust are well documented in the published scientific papers listed in Appendix B. It is of interest to note that the importance of research into the biochemistry of mental retardation was recognised at Stoke Park very early. By 1939 every new admission was being tested for phenylketonuria and other urine and blood abnormalities. In 1940 Mr. A. H. Tingey was appointed as full-time biochemist to the Burden Mental Research team.

Mrs. Burden, as the Chairman of the trustees of the Burden Trust, built at Stoke Park, at the suggestion of a surgeon, a clinic for surgical treatment of the patients in Stoke Park Colony. This idea was later abandoned and the Medical Research Council suggested that the premises be used as a neuro-research centre for the West of England. Mrs. Burden accepted the idea and gave further financial support and the *Burden Neurological Institute* was opened on 12th May, 1939. Professor F. L. Golla was appointed director of the Institute; Dr. W. Grey Walter was in charge of the Physiological Research Unit; Dr. E. L. Hutton was in charge of the Psychiatric Research; Mr. L. D. MacLeod and Mr. A. Tingey were appointed as biochemists and Professor Max Reiss was in charge of the Endocrinological unit. Two neuro-surgeons, Mr. McKissoch and Mr. Willway, gave their services.

The Institute became nationally and internationally known for its work, especially in the studies of electrophysiology of the C.N.S., electro-encephalography, and the physical treatment of mental disorder. Patients at the Stoke Park Group are benefiting greatly from all the latest advances at the Burden

Institute. Just before the second world war, the Burden Institute initiated the practice of electric convulsive therapy in this country, and soon after the first leucotomy in Great Britain was performed there (Walter, 1969). The Burden Institute remained outside the National Health Service until 1st April, 1968, when it became part of the Cosham/Frenchay Hospital Group. In 1940, Professor J. A. Berry retired and was succeeded by Dr. R. M. Bates as Medical Superintendent. Dr. Bates left Stoke Park in 1946, and Dr. R. M. Norman was appointed in his place.

The need for the care of old patients was already realised in 1940, when Coldharbour Farm was purchased and later converted for the use of geriatric female patients. The same year a property in Bristol, Anchor Lodge, was acquired and used as a hostel for high-grade girls who were working in various jobs in town.

Diet plays a very important role in the treatment of mentally retarded patients and a housekeeping sister-dietician was appointed in 1947. To implement the latest knowledge in the field of dietetics, a part-time dietician was appointed in 1968.

During 1945, facilities were given to Dr. A. Q. Wells, who was working for the Medical Research Council, to carry out an investigation on the subject of tuberculosis and its prevention, among the patients at Stoke Park Colony. In 1937, Dr. Wells had discovered a form of tuberculosis peculiar to voles, which has a very low virulence to man. By the inoculation of a suitable dosage of vole tubercle bacilli, it has been shown that the resistance to human and bovine tuberculosis may be raised in normal children who have not suffered previously from the disease. Recently the vole bacillus again received attention in the medical press (Brit. med. J., 1969).

In 1948, Stoke Park Colony was absorbed into the present National Health Service and *Stoke Park Hospital Group* came into being, consisting of Stoke Park, Purdown, Hanham Hall and Leigh Court Hospitals. Whittington Hall, originally acquired by Mr. Burden, became part of the hospitals for mentally retarded patients under the Sheffield Regional Hospital Board.

In 1953, Dr. R. M. Norman became Director of the Neuropathological Laboratory, Frenchay Hospital, Bristol, and was succeeded as Medical Superintendent at Stoke Park in 1954 by Dr. W. A. Heaton-Ward, who served in this capacity until 1963, when the post was redesignated Consultant Psychiatrist-in-Charge.

The foundations laid by Mr. Burden and the tradition of a multi-disciplinary approach to the problems of mental retardation became firmly established at Stoke Park, as is evident from Stoke Park Studies, Second Series (Jancar, 1961), from other publications and various projects, and from close co-operation with other hospitals in Bristol and elsewhere.

In 1956, premises owned by the Hospital Group in Clevedon, Somerset, were converted into a Holiday Home, where over 800 patients from the hospital have at least one week's holiday by the sea each year.

To the Group's well established occupational therapy departments, Industrial Therapy, in the form of contracts with outside firms was added in 1959, to further the rehabilitation of in-patients and day-patients. The experiment was very successful, especially with lower-grade patients (Cameron and Nicoll, 1961), and is expanding throughout the Group. Occupational therapy, industrial therapy, and the discovery of new drugs for the treatment of epilepsy, severe behaviour disorders, and psychotic episodes, changed the lives of in-patients and made them more receptive to further training and rehabilitation. Many were able to return to community care and work in outside jobs.

The importance of community care for the mentally retarded and the avoidance of hospital admission whenever possible, was always the aim at Stoke Park and in 1958 an assessment clinic was established in conjunction with the Bristol Local Health Authority. Another was opened in 1962, at the Gloucestershire Royal Hospital and in 1965, one was started at the Bush Training Centre in Bristol. It was soon found that complete assessment of some patients was not possible at the assessment clinics as the time required and the facilities for further investigations were not available. A new children's ward opened in 1959 helped greatly in the treatment of retarded children.

An Assessment Unit was opened in October 1961 in Hanham Hall Hospital, and a few of the beds in the newly opened sick ward were utilised for this purpose, when available. The success of the assessment unit soon became apparent and facilities were extended throughout the Stoke Park Group and patients of all ages and both sexes were admitted for further assessment, whenever a bed became available. Not only the patients, but also the relatives, the community, nursing and other ancillary staff, long-stay patients, and medicine as a whole benefited by this venture (Jancar, 1969). A 20-bedded Assessment Unit for children and adults of both sexes is being built at present at Stoke Park Hospital, with five other new wards to replace outdated buildings.

In 1961, a unit for disturbed adolescent females was opened in Coldharbour Farm, which became vacant when geriatric patients were moved to other hospital premises. The aim of the unit was to rehabilitate girls between about 15 and 25 years of age, with I.Q.'s of about 70 and over, whose extremely disturbed behaviour had failed to respond to various authoritarian regimes, which included approved schools and prisons. The method of the rehabilitation was to be, as far as possible, permissive and psychotherapeutically orientated.

Once a patient has achieved reasonable stability and satisfactory work habits within the unit, the social worker obtains suitable employment for her outside the unit. The range of employment includes domestic or laundry work in nearby general hospitals, hotels, restaurants, canteens and various jobs in offices and factories. After a period of satisfactory daily employment, attempts are made to find residential employment or lodging for patients (Heaton-Ward, 1969).

Teaching undergraduate and postgraduate students about mental retardation at Stoke Park is a long established tradition. It consists of lectures, clinical demonstrations, clinical meetings and lecture-courses for the Diploma of Psychological Medicine and other degrees and diplomas. At one of the clinical meetings, on 30th November, 1962, when Professor C. E. Dent was the guest speaker, the inaugural meeting of the Bristol Psychiatric Society took place. To further the knowledge and advances in the field of mental retardation, seminars for qualified nurses, teachers, occupational therapists, and medical auxilliary staff of the Stoke Park Group and other hospitals in Bristol, including local health authorities, have become annual events since 1959.

### RECENT ADVANCES IN THE STOKE PARK GROUP

Chromosomal Studies opened a new field in the investigation of the causation of mental and physical abnormalities. Professor L. S. Penrose from the Galton Laboratory, and later Dr. F. J. W. Lewis from Southmead Hospital, Bristol, undertook the chromosomal analysis of patients with various abnormalities at Stoke Park. The following chromosomal abnormalities have been detected:—Presumptive trisomy—16, presumptive mosaic—trisomy 16, ring chromosome 18, examples of trisomy 21, mosaic Down's syndrome, translocation Down's syndrome, OX, OX/XX, XXY, "male pseudohermaphroditism ", with XX, XXXY, XXXXY and XXYY.

Chromotography of urine and blood was performed soon after the method became known, by Professor C. E. Dent, University College, London, on a number of patients whose congenital abnormalities suggested a biochemical disorder. Dr. R. D. Eastham and his staff at Frenchay Hospital, Bristol, continued this analysis when the equipment was installed in that hospital. Other biochemical studies were undertaken by Stoke Park staff and Dr. Eastham's team. "Pink Spot" was detected in the urine of epileptic patients under treatment with promazine hydrochloride. Abnormal plasma viscosity in Down's syndrome was reported. Large studies on serum cholesterol were carried out with the help of the Mental Health Research Fund, which yielded valuable information about the normal and abnormal levels in Down's syndrome and in other mentally retarded patients of both sexes and all ages. Recently the red cells of patients with Down's syndrome, and of epileptics on long-standing anticonvulsive therapy, were measured and compared with the cells of normal and other retarded patients. The results revealed conclusive macrocytosis in patients with Down's syndrome and in epileptics on prolonged treatment.

Since scientific reappraisal of dermatoglyphic studies took place in the field of mental retardation, palm and foot prints have been collected at Stoke Park from patients suffering from congenital abnormalities and syndromes. The dermatoglyphs were analysed by Professor L. S. Penrose and Dr. Sarah Holt, Galton Laboratory, London.

Gas chromatography has enabled detailed studies of lipid metabolism to be carried out. Dr. R. W. R. Baker, Guy's Hospital, London, performed the first studies for Stoke Park and further studies have been undertaken recently by Dr. G. K. McGowan at Bristol Royal Infirmary.

Ultrasonic waves, as a useful adjunct in the diagnosis of congenital and acquired malformations, was introduced at Stoke Park in 1966 by Dr. Douglas Gordon from Moorfields Eye Hospital, London.

New techniques and latest appliances are used in dentistry, psychology, nursing, teaching, speech therapy, physiotherapy and other spheres to complement modern treatment and rehabilitation of the patients.

The efficient administration and maintenance of the hospitals with modern equipment is of great help to the well-being of the patients and the welfare of the staff. The dedicated and forward-looking members of the Management Committee, acting on behalf of the Regional Hospital Board, are the supporting influence in the running and development of the services in the hospitals to which over 10,000 patients had been admitted up to 1st April, 1969.

*Wardens and Chairmen of the Stoke Park Group Hospital Management Committee.*

*Warden*:
The Rev. H. N. Burden. 1909-1930.
Mrs. R. G. Burden. 1930-1939.
Lt.-Col. E. C. Brown. 1939-1946.

*Acting Warden*:
Dr. C. Visger, O.B.E. 1946-1948.

*Chairmen*:
Dr. C. Visger, O.B.E. 1948-1951.
Dr. H. J. Orr-Ewing. 1951-1953.
W. R. Gibbons, Esq., J.P. 1953-1968.
Alderman C. Hebblethwaite, C.B.E. 1968 to date.

*The Hospital League of Friends.*

In October, 1954, the Loyal Order of Moose sponsored the formation of the League of Friends. Since its foundation the members have greatly contributed, with their enthusiasm, devotion and fund raising activities, towards the happiness, rehabilitation and extra amenities for the patients throughout the hospital group.

### CONCLUSION

The span of 60 years in time means very little, but 60 years of Stoke Park are packed with pioneering work in diagnosis, treatment and prevention of mental retardation. The Founder's foresight in his multi-disciplinary approach in this field, which has remained at Stoke Park throughout its existence, was confirmed at five recent international congresses for mental retardation. It was appropriate that on 27th March, 1969, on the eve of the 60th anniversary of the foundation of the Stoke Park Group, the South Western Division and Mental Deficiency Section of the R.M.P.A. should hold its spring meeting at Stoke Park and that the Diamond Jubilee lectures should be delivered on the same day by the doyens in the field of mental research:—

| | |
|---|---|
| Professor L. S. Penrose, F.R.S. | —"Dermatoglyphics and Chromosomal Abnormalities in mental Subnormality". |
| Dr. J. A. Fraser Roberts, F.R.S. | —"Genetics in Mental Subnormality". |
| Dr. W. Grey Walter | —"Progress in the Correlation of Mentality and Brain Mechanism". |

Sixty years of Stoke Park is another chapter added to the history of the great work being done in Bristol for the treatment, care and prevention of mental illness, which started in 1696, when the Old Mint in Bristol was converted into St. Peter's Hospital, the first public mental hospital.

It is important that from time to time we should stop in our sometimes bewildered and confused world, and walk into the past among the shadows of great men and their work. We find there not only tranquillity and humility but also clarity, vision, and inspiration for our work in the future.

ACKNOWLEDGEMENTS

I am very grateful to Dr. W. A. Heaton-Ward, Consultant Psychiatrist-in-Charge, for helpful and constructive criticism of the paper; to Mr. B. F. Thompson, Group Secretary, for making available the relevant hospital records and photographs; to Josiah Wedgwood & Sons, Staffordshire, for the photograph and the history of the dinner plate depicting "Stoke Gilfort"; to Mr. L. Nott, East Bristol Evening Institute, for valuable historical information; to many members and retired staff for help with the data, reprints and personal knowledge about the past of the Hospital Group and to Mrs. K. J. Hiscock for the secretarial work.

REFERENCES

1. Berry, R. J. A. (1933) Mental Deficiency — Stoke Park Studies — First Series. MacMillan and Co. Ltd., London.
2. British Medical Journal (1969) Of Voles and Men (Leading article) 1, 527.
3. Heaton-Ward, W.A. (1969) Coldharbour Farm — The First Five Years. Bristol med.-chir. J. **84**, 46.
4. Jancar, J. (1961) Stoke Park Studies—Mental Subnormality—Second Series. John Wright & Sons Ltd., Bristol.
5. Jancar, J. (1969) Assessment Unit for the Mentally Retarded (A Six Years' Survey) (in press).
6. Norman, R. M. and Wood, N. (1941). A Congenital Form of Amaurotic Family Idiocy. J. Neurol. Psychiat. **4**, 175.
7. Walter, W. G. (1969) Personal communication.

APPENDIX A

BOOKS PUBLISHED BY MEDICAL STAFF

1. Berry, R. J. A. and Gordon, R. G. (1931) The Mental Defective — A Problem in Social Inefficiency. Kegan Paul, French, Trubner & Co. Ltd., London.
2. Berry, R. J. A. (1933) Mental Deficiency — Stoke Park Studies — First Series. MacMillan and Co. Ltd., London.
3. Berry, R. J. A. (1938) A Cerebral Atlas. Oxford University Press.
4. Berry, R. J. A. (1939) Your Brain and Its Story. Oxford University Press.
5. Roberts, J. A. F. (1940) An Introduction to Medical Genetics. Oxford University Press. (Fourth Edition, 1967).
6. Langan, Ivy W. (1945) Instruction Booklet for a special adaptation for the Blind of the 1937 revision of the Stanford-Binet Tests. J. W. Arrowsmith Ltd., Bristol.
7. Allison, D. R. and Gordon, R. G. (1948) Psychotherapy. Its Uses and Limitations. Oxford University Press.
8. Lyons, J. F. and Heaton-Ward, W. A. (1953) Notes on Mental Deficiency. John Wright and Sons Ltd., Bristol. (Third Edition, 1955).
9. Alexander, G. L. and Norman, R. M. (1960) The Sturge-Weber Syndrome. John Wright and Sons Ltd., Bristol.
10. Heaton-Ward, W. A. (1960) Mental Subnormality. John Wright and Sons Ltd., Bristol. (Third Edition, 1967).
11. Jancar, J. (1961) Stoke Park Studies — Mental Subnormality — Second series. John Wright and Sons Ltd., Bristol.
12. Eastham, R. D. and Jancar, J. (1968) Clinical Pathology in Mental Retardation. John Wright and Sons Ltd., Bristol.

### APPENDIX B
### PAPERS PUBLISHED BY MEDICAL STAFF

1930

1. Berry, R. J. A. Hospitals — voluntary or self-supporting? Bristol med.-chir. J. **47**, 19.
2. Berry, R. J. A. Child Guidance from the brain physiologist's point of view. Bristol med.-chir. J. **47**, 120.

1931

3. Berry, R. J. A. Cerebral Cortical Structure and its Relations to Mental Disease. Brit. med. J. **1**, 837.

1932

4. Berry, R. J. A. Brain Structure in Relation to the Mind. Illustrated by new and original models. J. Neurol. Psychopath. **13**, 97.
5. Berry, R. J. A. and Bates, R. M. A case of Porencephalic Imbecility. Brit. med. J. **1**, 830.
6. Berry, R. J. A. Mental Deficiency Pictorially Recorded. Brit. med. J. **2**, 807.
7. Berry, R. J. A. Mental Deficiency in England: An Analysis of the Mental, Physical and Medical Characteristics of a Group of 162 Adult Feeble-minded Women. Bristol med.-chir. J. **49**, 177.
8. Gordon, R. G. and Norman, R. M. Some Psychological Experiments with Mental Defectives. Brit. J. Psychol. **23**, parts 1 and 2.

1933

9. Berry, R. J. A. Unselected Examples of the Hereditary Transmission of Endogenous Amentia. Eugen. Rev. **24**, 285.
10. Gordon, R. G. Berry, R. J. A. and Norman, R. M. Neurological Abnormalities: Their Occurrence and Significance as Illustrated by an Examination of 500 Mental Defectives. J. Neurol. Psychopath. **14**, 97.
11. Gordon, R. G. The Merrill-Palmer Scale of Intelligence Test for Pre-School Children applied to low-grade Mental Defectives. Brit. J. Psychol. **24**, Part 2, October.

1934

12. Berry, R. J. A. The Problem of the Mental Defective. Proc. Health Congress of the Royal Sanitary Institute, Bristol, July.
13. Berry, R. J. A. and Norman, R. M. Cerebral Structure and Mental Function as illustrated by a study of four defectives' Brains, J. Neurol. Psychopath. **14**, 289.
14. Gordon, H. L. The Intentional Improvement of Backward Tribes. E. Afr. med. J. **11**, 143.
15. Gordon, H. L. Enquiry into Correlation of Civilisation and Mental Disorder in the Kenyan Native. E. Afr. med. J. **12**, 327.
16. Gordon, H. L. Certification of Mental Disorder in Kenya. E. Afr. med. J. **12**, 357.
17. Bates, R. M. A Note on the Value of the Dick Test for Institutional Purposes. Lancet **1**, 1006.
18. Berry, R. J. A. Some lesser known Views of Mental Deficiency. Mental Welfare, **15**, No. 2, April.

1935

19. Berry, R. J. A. Some of the Structural Abnormalities presented by the Brains of Thirty-one Certified Mental Defectives. J. Neurol. Psychopath. **16**, 54.
20. Norman, R. M. A Case of Juvenile Amaurotic Idiocy. J. Neurol. Psychopath. **15**, 219.
21. Gordon, R. G. and Norman, R. M. A Case of Acute Toxic Chorea. J. Neurol. Psychopath. **15**, 313.
22. Roberts, J. A. F., Norman, R. M. and Griffiths, Ruth. Studies on a Child Population: 1. Definition of the Sample, Method of Ascertainment and Analysis of the Results of a Group Intelligence Test. Ann. Eugen. **6**, 319.
23. Roberts, J. A. F. Hereditary and Mental Deficiency. Brit. med. J. **1**, 413.
24. Roberts, J. A. F. Twins. Eugen. Rev. **27**, 25.
25. Bates, R. M. Streptococcal Septicaemia treated with antitoxin. Lancet. **1**, 1154.

**1936**

26. Norman, R. M. Bilateral Atrophic Lobar Sclerosis following Thrombosis of the Superior Longitudinal Sinus. J. Neurol. Psychopath. **17**, 135.
27. Bates, R. M. Non-Spirochaetal Infectious Jaundice. Brit. med. J. **1**, 521.
28. Berry, R. J. A. Brain Size and Mentality. Brit. med. J. **2**, 62.
29. Berry, R. J. A. What to do with the Mental Defective in Private Practice. Medical Press and Circular No. 5089.

**1937**

30. Roberts, J. A. F. and Griffiths, Ruth. Studies on a Child Population: II. Re-tests on the Otis and Stanford-Binet Scales, with a Note on the use of a Shortened Binet Scale. Ann. Eugen. **8**, 15.
31. Norman, R. M. An example of Status Marmoratus of the Cerebral Cortex. J. Neurol. Psychiat. **1**, 1.
32. Bates, R. M. A Case of Naevoid Amentia. Lancet **1**, 1282.
33. Berry, R. J. A. The Burden Mental Research Trust; its Present and Future. Bristol med-chir. J. **54**, 201.
34. Roberts, J. A. F. Sex-linked Microphthalmia sometimes associated with Mental Deficiency. Brit. med. J. **2**, 1213.
35. Roberts, J. A. F. The Place of Genetics in the Practice of Medicine. Newcastle med. J. **17**, 115.
36. Roberts, J. A. F. and Perry, C. B. A Study on the Variability in the Incidence of Rheumatic Heart Disease within the City of Bristol. Brit. med. J. **2**, Suppl. 154.

**1938**

37. Berry, R. J. A. Cerebral Malformations and their Clinical Consequences. Bristol med-chir. J. **55**, 111.
38. Roberts, J. A. F., Norman, R.M. and Griffiths, Ruth. Studies on a Child Population, III. Intelligence and Family Size. Ann. Eugen. **8**, 178.
39. Roberts, J. A. F., Norman, R. M. and Griffiths, Ruth. Studies of a Child Population, IV. The Form of the Lower End of the Frequency Distribution of Stanford-Binet Intelligence Quotients and the Fall of Low Intelligence Quotients with Advancing Age. Ann. Eugen. **8**, 319.
40. Gordon, R. G. and Norman, R. M. Further Observations on Neurological Abnormalities in Mental Defectives. J. Neurol. Psychopath. **1**, 173.
41. Norman, R. M. Some Observations on the Depth and Nerve-cell content of the Supragranular Cortex in Normal and Mentally Defective Persons. J. Neurol. Psychopath. **1**, 198.
42. Gordon, R. G. and Roberts, J. A. F. Paraplegia Mongolism in Twins. Arch. Dis. Childh. **13**, 79.

**1939**

43. Roberts, J. A. F. Intelligence and Family Size. Eugen. Rev. **30**, 237.
44. Roberts, J. A. F. Observations of a Representative Group of Children of School Age, with an account of some Family and Social Characteristics of the Brightest the Average and the Dullest. Proc. Amer. Ass. ment. Defic. **44**, 79.
45. Gordon, R. G., Fraser, J. A. F. and Griffiths, Ruth. Does Poliomyelitis Affect Intellectual Capacity? Brit. med. J. **2**, 803.
46. Iles, A. E. Recessive Sex-linked Blindness. Proc. roy. Soc. Med. **32**. 1614.
47. Berry, R. J. A. An Investigation into Mental State of the Parents and Sibs of 1,050 Mentally Defective Persons. Bristol med.-chir. J. **56**, 189.
48. Berry, R. J. A. Some of the Social Aspects of Mental Deficiency in the Wage-earning Classes. Brit. med. J. **1**, 332.
49. Bates, R. M. A Case of Cutaneous and Conjunctival Diphtheria. Brit. J. Derm. Syphil. **51**, 76.
50. Bates, R. M. Renal Dwarfism. Brit. J. Child. Dis. **35-36**, 34.
51. Bates, R. M. Three cases of Phenylpyruvic Oligophrenia. J. ment. Sci. **85**, 273.

**1940**

52. Norman, R. M. Nerve-cell Swelling of the Juvenile Amaurotic Family Idiocy Type. Arch. Dis. Childh. **15**, 244.
53. Norman, R. M. Cerebellar Atrophy Associated with Etat Marbré of the Basal Ganglia. J. Neurol. Psychiat. **3**, 311.

54. Norman, R. M. and Taylor, A. L. Congenital Diverticulum of the Left Ventricle of the Heart in a case of Epiloia. J. Path. Bact. **50**, 61.
55. Whitnall. S. E. and Norman, R. M. Microphthalmia and the Visual Pathways. A Case Associated with Blindness and Imbecility and sex-linked. Brit. J. Ophthal. **24**, 229.
56. Norman, R. M. Primary Degeneration of the Granular Layer of the Cerebellum: An Unusual Form of Familial Cerebellar Atrophy Occurring in Early Life. Brain. **63**, 365.
57. Roberts, J. A. F. Surnames, Intelligence and Fertility. Nature, **145**, 939.
58. Roberts, J. A. F. Intelligence and Fertility. Ment. Hlth. **1**, 69.
59. Roberts, J. A. F. Studies on a Child Population. V. The Resemblance in Intelligence Between Sibs. Ann. Eugen. **10**, 203.

**1941**

60. Norman, R. M. and Wood, N. A Congenital Form of Amaurotic Family Idiocy. J. Neurol Psychiat. **4**, 175.
61. Roberts, J. A. F. Inheritance of Mental Deficiency. Proc. Seventh International Genetical Congress. Cambridge University Press. p. 249.
62. Roberts, J. A. F. The Negative Association between Intelligence and Fertility. Hum. Biol. **13**, 410.

**1942**

63. Fleming, G. W. T. H. and Norman, R. M. Arhinencephaly with Incomplete Separation of the Cerebral Hemispheres. J. ment. Sci. **88**, 341.
64. Roberts, J. A. F. Surnames and Blood-Groups with a note on a Probable Remarkable Difference Between North and South Wales. Nature, **149**, 138.
65. Roberts, J. A. F. Blood-Group Frequencies in North Wales. Ann. Eugen. **11**, 260.
66. Roberts, J. A. F. Blood-Group Frequencies in South-Western England and North Wales: A Study in Racial Variation, together with a search for Evidence that the Blood-Groups possess selective value. M. D. Thesis, University of Edinburgh.

**1943**

67. Roberts, J. A. F. and Fisher, R. A. A Sex Difference in Blood-Group Frequencies. Nature, **151**, 640.

**1944**

68. Norman, R. M. Atrophic Sclerosis of the Cerebral Cortex Associated with Birth Injury. Arch. Dis. Childh. **19**, 111.
69. Roberts, J. A. F. Intelligence and Season of Conception. Brit. med. J. **1**, 230.
70. Roberts, J. A. F. Intelligence and Season of Conception. Brit. med. J. **1**, 539.

**1945**

71. Roberts, J. A. F. On the Difference between the Sexes in Dispersion of Intelligence. Brit. med. J. **1**, 727.
72. Curran, D. and Roberts, J. A. F. A Screening Procedure for the Selection of Recruits for Psychiatric Interview. J. ment. Sci. **41**, 290.
73. Norman, R. M. Thalamic Degeneration following Bilateral Premotor Frontal Lobe Atrophy of the Strümpell Type J. Neurol. Neurosurg. Psychiat. **8**, 52.
74. Roberts, J. A. F. Genetic Linkage in Man, with particular reference to the usefulness of very small bodies of data. Quart. J. Med. **14**, 27.

**1946**

75. Roberts, J. A. F. Racial and Geographical Variations in the Frequencies of the Blood Groups. J. roy. nav. med. Serv. **32**, 187.

**1947**

76. Roberts, J. A. F. High Grade Mental Deficiency in relation to Differential Fertility. J. ment. Sci. **43**, 289.
77. Fairweather, D. S. and O'Sullivan, H. J. L. Gastric Dilatation, Megacolon and Idiocy in Identical Twins. Arch. Dis. Childh. **22**, 236.
78. Norman, R. M. Diffuse Progressive Metachromatic Lencoencephalopathy. Brain. **70**, 234.

79. Norman, R. M. Etat Marbré of the Corpus Striatum following Birth Injury. J. Neurol. Neurosurg. Psychiat. **10**, 12.
80. Roberts, J. A. F. The Inheritance of the Rh. Blood-Group. Med. Press. **217**, 7.
81. Roberts, J. A. F. Birth Order, Maternal Age and Intelligence. Brit. J. Psychol. **1**, 35.

**1948**

82. Norman, R. M. Cerebral Diplegia following Birth injury. Bristol med-chir. J. **65**, 43.
83. Roberts, J. A. F. The frequencies of the ABO Blood Groups in South Western England. Ann. Eugen. **14**, 109.
84. Roberts, J. A. F. Return of Sickness from Ships of the Royal Navy (1945-46). A Contribution to Medical Climatology. Brit. J. soc. Med. **2**, 55.
85. Roberts, J. A. F., Jennison, R. F. and Penfold, J. B. An Application to a Laboratory Problem of Discriminant Function Analysis Involving more than Two Groups. Brit. J. soc. Med. **2**, 130.

**1949**

86. Asher, Cécile and Roberts, J. A. F. A Study on Birthweight and Intelligence. Brit. J. Soc. Med. **3**, 56.
87. Norman, R. M. Etat Marbré of the Thalamus following Birth Injury. Brain **72**, 83.
88. Fairweather, D. S., O'Sullivan, H. J. L. and Walter, W. G. Unverricht's Myoclonic Epilepsy in Identical Twins. E.E.G. Clin. Neurophysiol. **1**, 115.
89. Roberts, J. A. F., Jennison, R. F. and Penfold, J. B. The value of Antigen in Wasserman reaction. J. clin. Path. **2**, 129.
90. Roberts, J. A. F. Blood Groups and Human Genetics. The Advancement of Science. **5**, 305.

**1950**

91. Norman, R. M. The Neuropathology of Oligophrenia. Recent Progress in Psychiatry. J. ment. Sci. **2**, 324.
92. Roberts, J. A. F. The Genetics of Oligophrenia. Proc. Congrès International de Psychiatrie, Paris, 1950.

**1951**

93. Sorsby, A., Brain, R. T. and Roberts, J. A. F. Essential Shrinking of the Conjunctiva in a hereditary affection allied to epidermolysis bullosa. Documenta Ophthalmologica. **5-6**, 118.

**1952**

94. Norman, R. M. Discussion on the Mental Deficiencies. Proc. 1st Congr. of Neuropathology 8-13 Sept. Rome. (Galton Lecture).
95. Roberts, J. A. F. The Genetics of Mental Deficiency. Eugen. Rev. **44**, 71.
96. Roberts, J. A. F. and Mellone, M. A. On the Adjustment of Terman-Merrill I.Q.s to secure comparability at Different Ages. Brit. J. Stat. Psychol. **5**, 65.

**1953**

97. Norman, R. M. The Pathology and Aetiology of Infantile Cerebral Palsies. Proc. roy. Soc. Med. **46**, 627.
98. Blacketer-Simmonds, D. A. An Investigation into the supposed differences existing between Mongols and other Mentally Defective Subjects with regard to certain Psychological Traits. J. ment. Sci. **99**, 702.
99. Dunsdon, M. I. and Roberts, J. A. F. The Relation of the Terman-Merrill vocabulary test to Mental Age in a sample of English Children. Brit. J. Stat. Psychol. **6**, 61.
100. Roberts, J. A. F. The use of Regressions Involving Variances of Dependent Variates for Calculating Age-corrected Scores. Biometrics, **9**, 267.
101. Roberts, J. A. F. An Analysis of the ABO Blood-Group. Records of the North of England. Heredity. **7**, 361.
102. Aird, I., Bentall, H. H. and Roberts, J. A. F. Relationship between Cancer of the Stomach and the ABO Groups. Brit. med. J. **1**, 799.

103. Dunsdon, M. I. A Comparison of Terman-Merrill Scale Test Responses among large sample of Normal, Maladjusted and Backward Children. J. ment. Sci. **99,** 720.
104. Roberts, J. A. F. Sex linked genes in man. Edinburgh Med. J. 60, 265.
105. Roberts, J. A. F. The Teaching of Medical Genetics. Proc. 1st World Conference on Med. Education. p. 652.

**1954**

106. Roberts, J. A. F. The Genetics of the Blood Groups. Postgrad. med. J. **30,** 58.
107. Hamilton, M., Pickering, G. W., Roberts, J. A. F. and Sowry, G. S. C. The Aetiology of Essential Hypertension. 1. The arterial pressure in the General Population. Clin. Sci. **13,** 11.
108. Hamilton, M., Pickering, G. W., Roberts, J. A. F. and Sowry, G. S. C. The Aetiology of Essential Hypertension, 2. Scores for Arterial Blood Pressure adjusted for differences in Age and Sex. Clin. Sci. **13,** 37.
109. Hamilton, M., Pickering, G. W., Roberts, J. A. F. and Sowry, G. S. C. The Aetiology of Essential Hypertension. 3. The Effects of Correcting for arm circumference on the Growth rate of arterial pressure with age. Clin. sci. **13,** 267.
110. Hamilton, M., Pickering, G. W., Fraser, J. A. F. and Sowry, G. S. C. The Aetiology of Essential Hypertension. 4. The Role of Inheritance. Clin. Sci. **13,** 273.
111. Aird, I., Bentall, H. H., Mehigan, J. A. and Roberts, J. A. F. The Blood Groups in Relation to Peptic Ulceration and Carcinoma of Colon, Rectum, Breast and Bronchus. An association between the ABO Groups and Peptic Ulceration. Brit. med. J. **2,** 315.
112. Roberts, J. A. F. The Relationship of he ABO Blood Groups to Cancer. Acta Un. int. Cancr. **10,** 155.

**1955**

113. Dunsdon, M. I. and Roberts, J. A. F. A Study of the Performance of 2,000 Children on Four Vocabulary Tests. a. Growth Curves and Sex Differences. Brit. J. Stat. Psychol. **8,** 3.
114. Weller, S. D. V. and Norman, R. M. Epilepsy due to Birth Injury in One of Identical Twins. Arch. Dis. Childh. **30,** 453.
115. Aird, I., Bentall, H. H. and Roberts, J. A. F. ABO Blood Groups and hypertension. Brit. med J. **2,** 321.
116. Roberts, J. A. F. Cousin Marriage (Long Fox Memorial Lecture). Med. J. of the South-West. **70,** 142.
117. Roberts, J. A. F. The ABO Blood Group and Disease. Proc. roy. Soc. Med. **48,** 143.

**1956**

118. Annett, J. and Kay, H. Skilled Performance. Occup. Psychol. **30,** 112.
119. Higgins I. T. T., Oldham, P. D., Merrick, A. J. and Dunsdon, M. I. Selection of Miners: A Survey of School-leavers in a Valley in South Wales. Brit. J. prev. and soc. Med. **10,** 32.
120. McConnell, R. B., Pyke, D. A. and Roberts, J. A. F. Blood groups in diabetes mellitus. Brit. med. J. **1,** 772.
121. Dickins, A. M., Richardson, J. R. E., Pike, L. A. and Roberts, J. A. F. Further observations on ABO blood group frequencies and toxemia of pregnancy. Brit. med. J. **1,** 776.
122. Roberts, J. A. F. (with a group of collaborators). An association between blood group A and pernicious anaemia. Brit. med. J. **2,** 723.
123. Roberts, J. A. F. Association between blood groups and disease. Advancement of Science. **51,** 191.

**1957**

124. Annett, J. The Information capacity of young mental defectives in an Assembly Task. J. ment. Sci. **103,** 621.
125. Dunsdon, M. I. and Roberts, J. A. F. A Study of the Performance of 2,000 Children on Form Vocabulary Tests. b. Norms, with some observations on the relative variability of Boys and Girls. Brit. J. Stat. Psychol. **10,** 1.
126. Roberts, J. A. F. ABO blood groups and duodenal ulcer. Brit. med. J. **1,** 75[illegible]

**1958**

127. Heaton-Ward, W. A. and Jancar, J. A Controlled Clinical Trial of Meprobamate in the Management of Difficult and Destructive Female Mental Defectives. J. ment. Sci. **104**, 454.

**1959**

128. Heaton-Ward, W. A. and Jancar, J. Promazine (Sparine) in the Treatment of Severe Behaviour Disorders of Mental Defectives. J. Mid. Men. Defic. Soc. 5, 43.
129. Heaton-Ward, W. A., Carpenter, W. H. K. and Jancar, J. Appearance of Parkinsonism in Mentally Defective Patients, treated with Dartalan, with the Occurrence of Oculogyric Crisis in Identical Twins. Bri. med. J. **2**, 407.
130. Norman, R. M., Urich, H. and Heaton-Ward, W. A. Neuropathological Findings in a case of Juvenile General Paresis treated with Penicillin. Brit. J. Vener. Dis. 35, 231.

**1961**

131. Heaton-Ward, W. A. Treatment of the Mentally Subnormal in Hospital. Proc. Conf. National Ass. Ment. Health. 53.
132. Jancar, J. Postencephalitic Endocrine Disorders with Mental Subnormality. J. Ment. Defic. Res., **5**, 115.

**1962**

133. Heaton-Ward, W. A. An Interim Report on a Controlled Trial of Niamid on the Mental Age and Behaviour of Mongols. Proc. London Conf. Scientific Study Mental Deficiency, London, **1**, 319.
134. Jancar, J. Mandibulo-Facial Dysostosis (Berry-Franceschetti Syndrome) associated with Severe Mental Subnormality and Consanguinity. Proc. London Conf. Scientific Study Mental Deficiency, London, **1**, 329.
135. Heaton-Ward, W. A. Healing and Psychiatry. J. C. of E. Hosp. Chaplain's Fellowship. 12.
136. Heaton-Ward, W. A. Interference and Suggestion in a Clinical Trial. J. ment. Sci. **108**, 865.
137. Jancar, J. Melleril and Placebo in the Treatment of Severely Subnormal Patients. J. ment. Subnorm. **8**, 52.

**1963**

138. Heaton-Ward, W. A. Psychopathic Disorder. Lancet, **1**, 121.
139. Jancar, J. Rickets with Secondary Hyperparathyroidism in a Severely Subnormal Child. Arch. Dis. Childh. **38**, 412.

**1964**

140. Heaton-Ward, W. A. A preliminary Report of a Case of Progeria associated with Mental Defect and an Hitherto Unreported Chromosome Abnormality. Proc. Int. Copenhagen Congr. on the Scientific study of Mental Retardation. **2**, 803.
141. Jancar, J. Mentally Defective Males with XXXXY Chromosomes. Proc. Int. Copenhagen Congr. for the Scientific Study of Mental Retardation. **1**, 179.
142. Lewis, F. J. W. and Jancar, J. Presumptive Translocation between a "21" Chromosome and one of the 6—12+X Group. The Human Chromosome. Newsletter. 12, 9.

**1965**

143. Eastham, R. D. and Jancar, J. Plasma Viscosity in cases of Severe Mental Subnormality. Amer. J. ment. Defic. **69**, 502.
144. Jancar, J. Cerebro-Metacarpo-Metatarsal Dystrophy (Pseudo-Pseudo-Hypoparathyroidism) with Chromosomal Anomaly. J. med. Genet. **2**, 32.
145. Heaton-Ward, W. A. The Problem of the Psychopath. The Practitioner, 194, 621.
146. Jancar, J. The Use of Haloperidol in the Treatment of Severe Behaviour Disorders in Mental Deficiency. Clin. Trials **J. 2**, 154.
147. Jancar, J. and Philpot, G. R. Porphobilinogen-like Chromogens in Urine of Epileptics. Brit. med. J. **1**, 1498.
148. Eastham, R. D., Jancar, J. and Duncan, E. H. L. Plasma Viscosity in Mental Deficiency and Down's Syndrome. Brit. J. Psychiat. **111**, 999.
149. Jancar, J. Rubinstein-Taybi's Syndrome. J. ment. Defic. Res. **9**, 266.

1966
150. Heaton-Ward, W. A. The Present Position of the Use of Tranquillisers on Psychiatric Patients. Current Medicines and Drugs. **6**, 14.
151. Jancar, J. Inverse Jaw-Winking with Exaggerated Bell's Phenomenon ("Ocular Stammer"). Ophthalmologica, Basel. **151**, 548.
152. Jancar, J. Hallerman-Streiff-François Syndrome (Dyscephalia Mandibulo-Oculo-Facialis). J. ment. Def. Res. **10**, 255.

1967
153. Heaton-Ward, W. A. Industrial Therapy for Severely Subnormal Patients in Hospital. Proc. IVth Int. Congr. of the World Fed. of Occupational Therapists in London. 1966. p. 95.
154. Jancar, J. Ectrodactyly, Spastic Paraplegia and Mental Retardation. J. ment. Defic. Res. **11**, 207.

1968
155. Heaton-Ward, W. A. The Expectation of Life of Mentally Subnormal Patients in Hospital. Proc. 1st Congr. of Int. Ass. for the Scientific Study of Mental Deficiency, Montpellier. p. 939.
156. Eastham, R. D. and Jancar, J. Serum Cholesterol in Mental Retardation. Proc. 1st Congr. of Int. Ass. for the Scientific Study of Mental Deficiency, Montpellier. p. 948.
157. Jancar, J. Naevus Syringocystadenomatosus Papilliferus. Proc. 2nd Int. Congr. of Neuro-Genetics and Neuro-Ophthalmology of the World Federation of Neurology. Montreal, 1967.
158. Heaton-Ward, W. A. The Need for a Comprehensive Service for the Mentally Subnormal. The Hospital. **64**, 135.
159. Jancar, J. XXYY with Manic Depression. Lancet. **2**, 970.

1969
160. Jancar, J. Paroxysmal Tachycardia, Epilepsy, Fragilitas Ossium and Mental Retardation. Bristol med.-chir. J. **84**, 17.
161. Heaton-Ward, W. A. Coldharbour Farm — The First Five Years. Bristol med.-chir. J. **84**, 46.
162. Jancar, J. Potter's Syndrome with Mental Retardation. (Aurorenal syndrome; Reno-facial Dysplasia). J. ment. Defic. Res. **13**, 8.
163. Eastham, R. D. and Jancar, J. Serum Cholesterol in Mental Retardation. Brit. J. Psychiat. **115**, (in press).
164. Eastham, R. D. and Jancar, J. Macrocytosis in Down's Syndrome. Lan. **1**, 895.
165. Jancar, J. Assessment Unit for the Mentally Retarded. (A Six Years' Survey). Proc. VIIth Int. Congr. on Mental Health, London, 1968. (In press).
166. Heaton-Ward, W. A. The Demand for Psychiatrists in Mental Subnormality. Conference on Postgraduate Psychiatric Education. The Training of Psychiatrists. (In press).
167. Jancar, J. Pellagra-Like Reaction due to Anti-Tuberculosis Treatment in a Subnormal. J. ment. Subnorm. (In press).

### APPENDIX C.

**Papers published by Nursing and Other Staff.**

1. Cooke, W. E. (1956) Speech Therapy and how it works. Health Horizon. p. 46.
2. Curtis, J. (1958) Oesophageal Carcinoma with Persistent Low Blood Pressure. Nursing Mirror. p. 10.
3. Curtis, J. (1959) A Student Nurse in a Mental Deficiency Hospital. Nursing Times. p. 793.
4. Cameron, F. and Nicoll, S. (1961) Industrial and Social Therapy; an Experiment. Nursing Times.
5. Cook, W. E. (1962) Speech Hindrance and Personality Reaction. Abstracts XIIth Congres: International Association of Logopedics and Phoniatrics. p. 19.
6. Peters, J. J. (1962) Two cases of Klippel-Feil Syndrome associated with Severe Mental Subnormality. Radiography. **28**, 316.
7. Whittaker, S. (1964) The Nursing Care of Subnormal Children. Nursing Mirror. **119**. 7.

8. Sampson, G. R. (1964) Fallacies on Purchasing. British Hospital and Social Services Journal.
9. Allen, A. E. (1965) The Principles of Nursing Applied to Everyday Care of the Mentally Subnormal. Nursing Times. **61**, 131.
10. Prescott, F. (1967) A Note on the Treatment of Mentally Handicapped Children by Means of Physiotherapy. The British Journal of Physiotherapy. **18**, 5.
11. Allen, A. E. (1967) Mind and Body. Nursing Times. **63**, 1181.
12. Prescott, F. (1968) World of Problems in Rehabilitation of the Disabled. The British Journal of Physiotherapy. **20**, 14.
13. Thompson, B. F. (1968) Problems of a Comprehensive Service. The Hospital. **64**, 135.
14. Pounds, V. A. (1968) The Severely Distressed Subnormal Child. Admission and Observation. Nursing Times. **64**, 226.
15. Pounds, V. A. (1968) Your Subnormal Child in Hospital? Nursing Times **64**, 478.
16. Pounds, V. A. (1968) Ward Management in a Subnormality Hospital. Nursing Times. **64**, 1104.
17. Hodges, B. E. (1968) The Future of Mental Subnormality Hospitals. Nursing Mirror. **126**, 22.
18. Pounds, V. A. (1968) Our Mental Subnormality Hospitals. Nursing Mirror. **126**, 34.
19. Campbell, C. M. (1968) Stereotyped and Expressive Movements in Imbeciles. Amer. J. ment. Defic. **73**, 195.

### APPENDIX D

### *Historical Notes on the Hospital Buildings.*

*Stoke Park Hospital*: *Dower Ward*. The first mention of Stoke in historical records is in the Domesday Book. This records that "*Duns* a Thane held *Stoche* in Ledbury hundred in the reign of Edward the Confessor . . . " After the Norman invasion of 1066, the Saxon Duns was dispossessed and the Manor of Stoche was given by William the Conqueror to one of his lieutenants, *Osborne Giffard*, who came from Scie in Normandy. His family had been known as the Lords of Longueville-la-Giffard. After the death of John, the last Giffard, the Manor of Stoke Gifford passed into the hands of Maurice de Berkeley, who became the founder of the Stoke Gifford branch of the family. The Manor of Stoke Gifford (Dower House of today) was rebuilt in 1760-1764, with the motto "Mihi Vobisque" (Mine and Yours), by Norborne Berkeley who was in 1768 appointed the Governor of Virginia. He died in the U.S.A. in 1770. The Manor of Stoke Gifford passed through his sister Elizabeth, who was by then the Duchess Dowager of Beaufort, to the Beauforts, and was for a long time used as a Dower House to Badminton. The 10th Duke of Beaufort sold the Manor in 1907 to the Rev. Burden.

Stoke Gifford was depicted in 1774 by an artist who painted the English scene for the Wedgwood Pottery, for an order of 952 pieces of dinner and dessert service for Empress Catherine II of Russia. This service is now on display at the Winter Palace in Leningrad (Plate XIX).

#### REFERENCES

1. Evans, D. R. (1958) A Short History of Stoke Giffort and its Parish Church. H. E. Iles. " The Central Press ", Kingswood, Bristol.
2. The Wedgwood Story (1967) Educational leaflet—Josiah Wedgwood & Sons Ltd., Barlaston, Stoke-on-Trent.

*Purdown Hospital* (Plate XX)

*Heath House* The first known deed relating to Heath House is dated 1425. Heath House belonged to the religious order in Bristol known as the Hospital of St. Bartholomew. Just before the dissolution of the monasteries by Henry VIII, Heath House was sold to Robert Thorne, a merchant of Bristol. In 1546, the Walters family became tenants of the house and their successors lived there for five generations. In 1767, through marriage, the Smyth family of Ashton Court, Bristol, became owners of Heath House until 1911, when it was bought by the Rev. H. N. Burden.

*Stapleton Grove* (Beech House). Stapleton Grove was built by Joseph Harford in about 1763. He was Sheriff of Bristol in 1779, Lord Mayor Elect in 1794, and a close friend of Edmund Burke. Henry Charles Harford sold Stapleton Grove to the Castle family in 1832. After the death of Mr. and Mrs. Castle in 1833, and on the invitation of Mary Carpenter, Rajah Ram Mohun Roy, an Indian religious and social leader and founder of Brahma Somaj, stayed in Stapleton Grove until he died in September 1833. He was buried in the Stapleton Grove grounds and ten years later interred at Arnos Vale Cemetery. After that the house was occupied by the Rector of Stapleton Church, Bishop Morell, and was also at one time used as a boys' school.

REFERENCES

1. Dahl, L. H. (1934) History of Stapleton, M.S.S.
2. Pountney, W. J. (1920) Old Bristol Potteries, J. W. Arrowsmith Ltd., Bristol.

*Leigh Court Hospital.* Leigh Court was called Lege in the Domesday Book. It records—" Turstin holds Lege, his father held it in King Edward's time and paid gheld for one hide . . . " The Manor of Leigh at one time belonged to the Monastery of St. Augustine at Bristol. At the Conquest, the Manor was given to the Bishop of Contanus, after whose death William Rufus granted the Manor to Robert FitzHammon, whose daughter married Robert Earl of Gloucester. The Earl of Gloucester sold the Manor of Leigh to Robert FitzHarding, who in 1148 bestowed it to the Abbey of St. Augustine, which he had founded in Bristol. After the dissolution of the Monastery in 1583, it was passed to Paul Bush the first Bishop of Bristol, and afterwards by grant of the King to Sir George Norton. On 16th September 1651 the Nortons gave shelter for four nights to King Charles II, after his defeat at Worcester. The property passed by marriage to the Trenchard family and it was purchased in the early 19th century by Mr. Philip John Miles who, between 1814 and 1816, built the Leigh Court of today. In January 1884 the late King Edward VII, then Prince of Wales, was entertained by the late Sir Philip Miles at Leigh Court.

"*Gethin Shilling.*" Dame Grace Gethin, wife of Sir Richard Gethin, was the last surviving member of the Norton family from Leigh Court. As benefactress, she is remembered by the bestowal of the " Gethin Shilling " on a number of widows at Westminster Abbey in a Lenten ceremony.

In the south aisle of Westminster Abbey stands an elaborate memorial to Grace Gethin, erected by her parents. In 1700 a book under the title " Reliquiae Gethinanae " was published and is preserved in the North library of the British Museum.

REFERENCES

1. Barnes, M. (1965) Abbots Leigh. Evening Post, Bristol, August 25th, p. 14.
2. Taylor, H. A. (1967) A Young Lady with a Marble Book. Country Life, March 23rd, p. 644.

*Hanham Hall Hospital.* (Plate XXII) Hanham Hall was built in 1655 by Richard Jones. On his death in 1697, it became the property of Thomas Tyre. Hanham Hall changed ownership again in 1791 and in 1803, when it was purchased by the Whittuck family, who stayed there until 1916. The finest feature of Hanham Hall is a very good example of an early 18th century shell-headed main entrance with flanking niches.

REFERENCE

1. Ellacombe, H. T. (1881) The History of the Parish of Bitton. William Pollard, North Street, Exeter.

Plate XIX

*'Stoke Gilford'* (1774) *Dower House*

Plate XX

*Purdown Hospital, Stapleton*

Plate XXI

*Leigh Court Hospital, Abbot's Leigh*

Plate XXII

## Figures and Tables

**Plate XIV f1:**
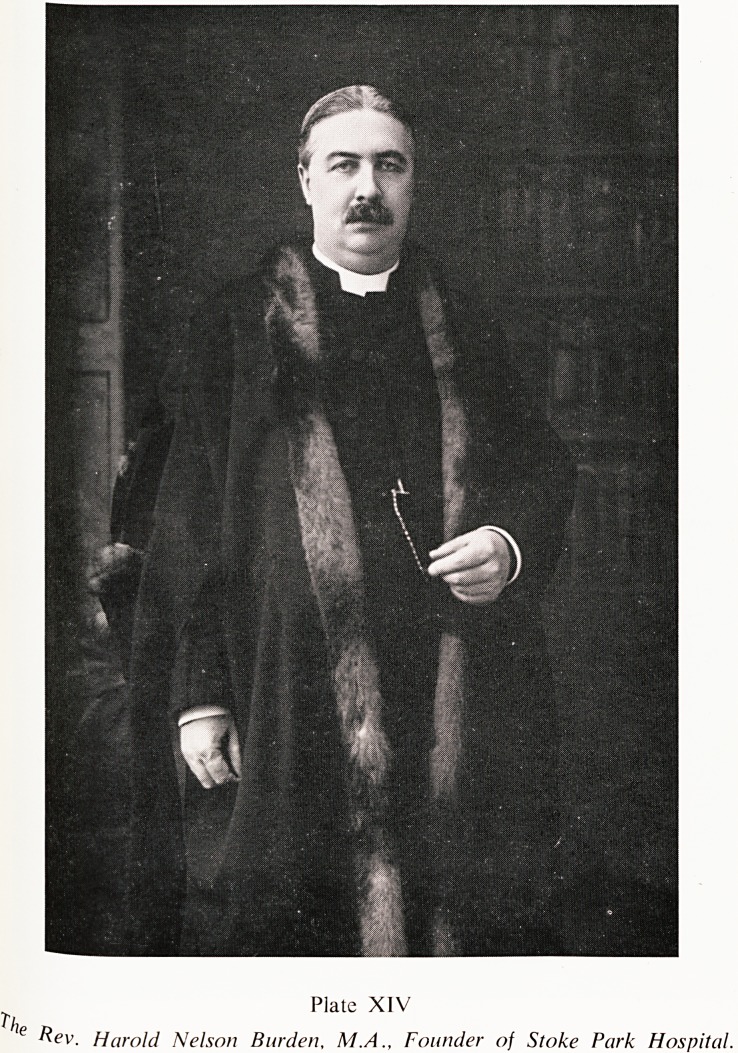


**Plate XV f2:**
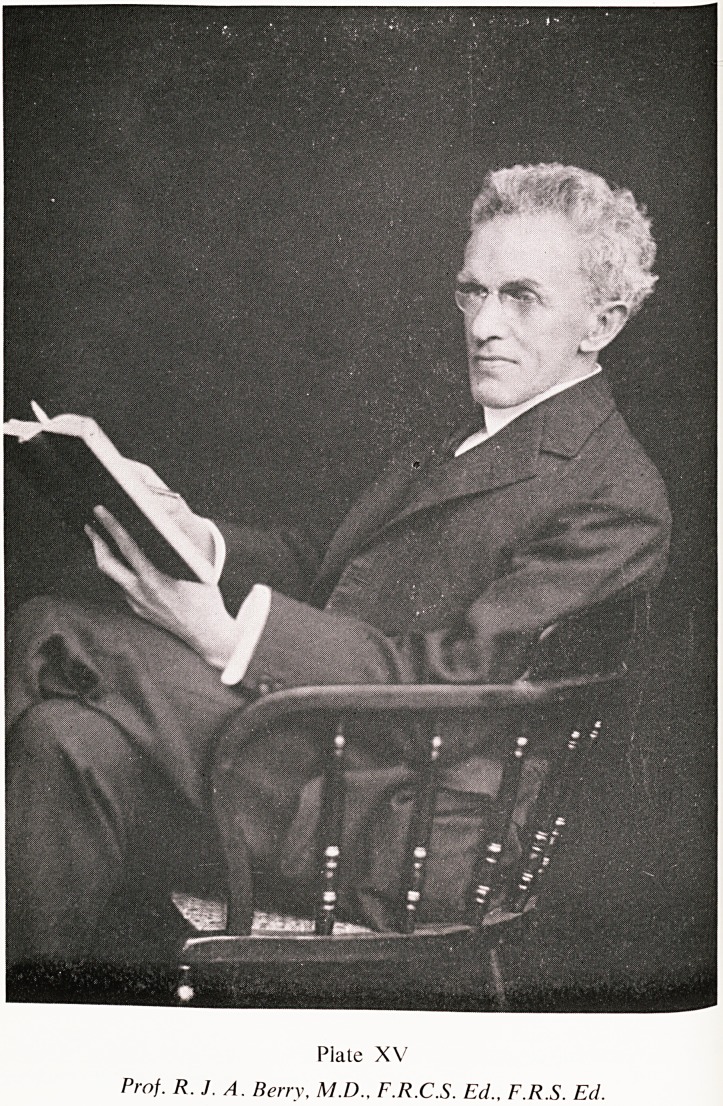


**Plate XVI f3:**
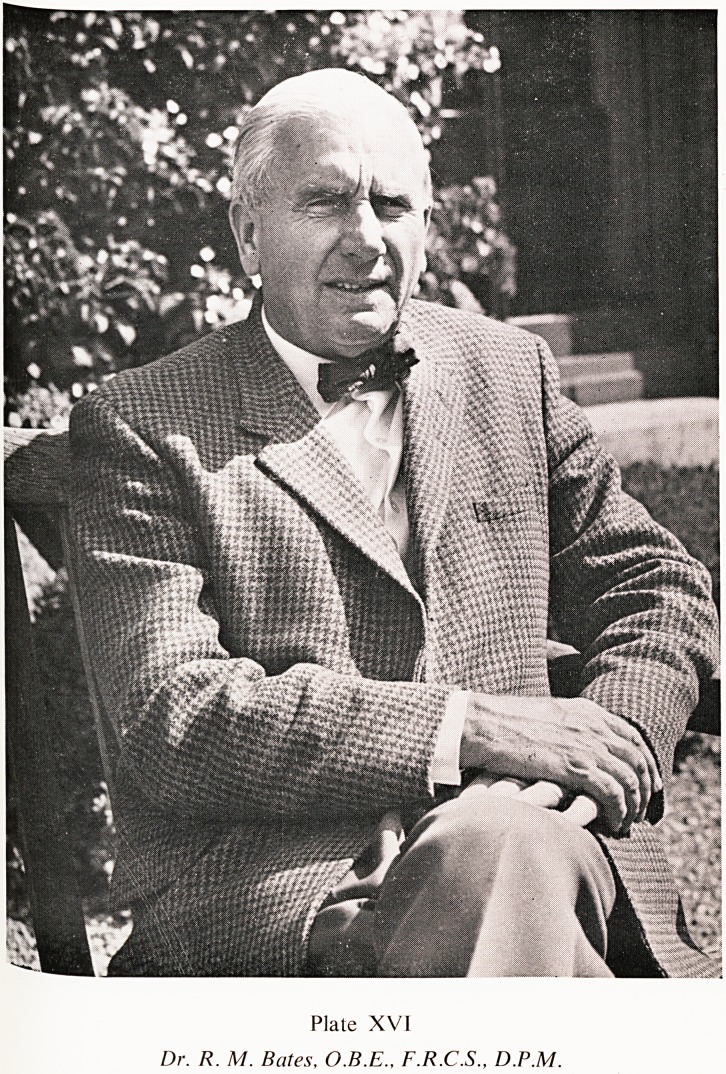


**Plate XVII f4:**
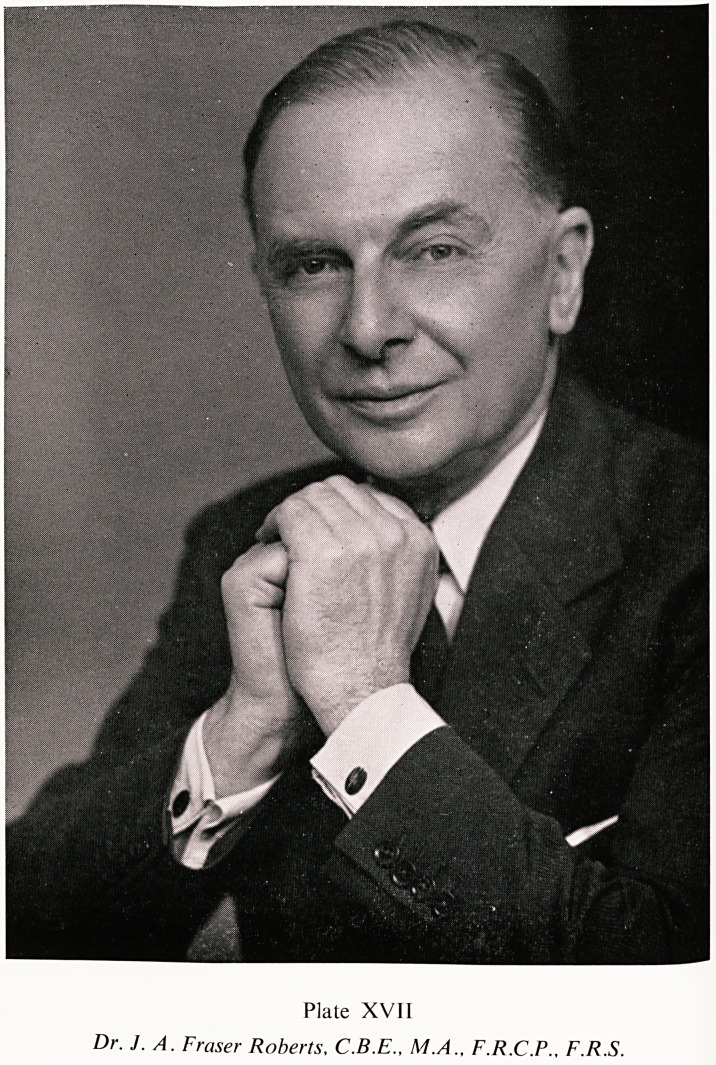


**Plate XVIII f5:**
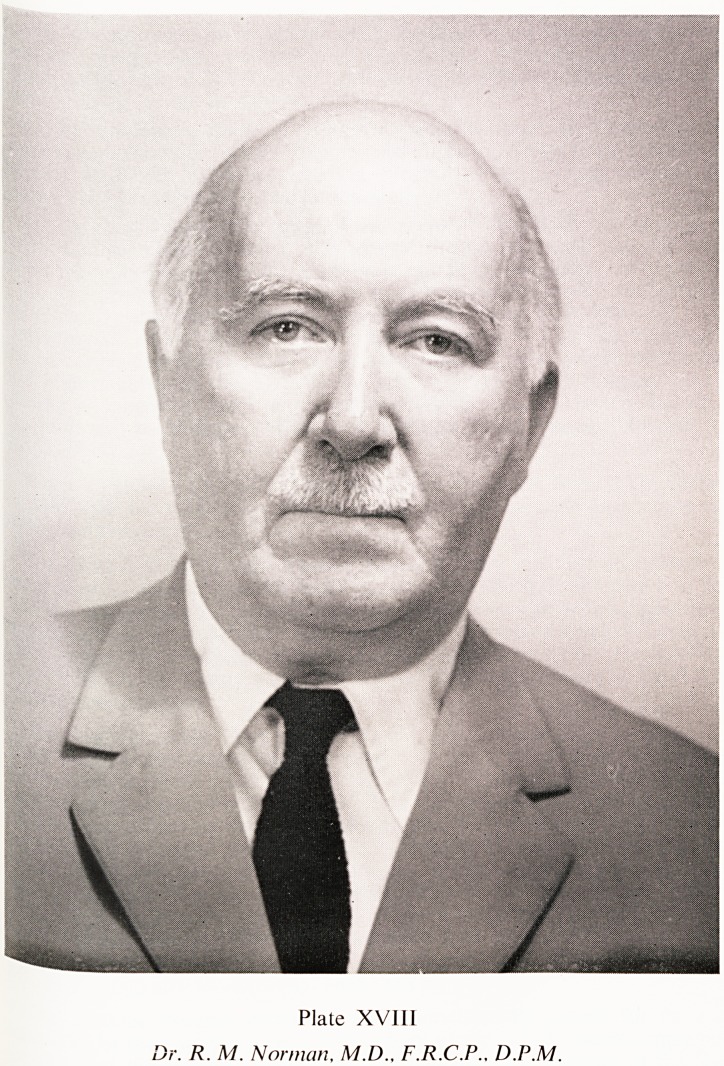


**Plate XIX f6:**
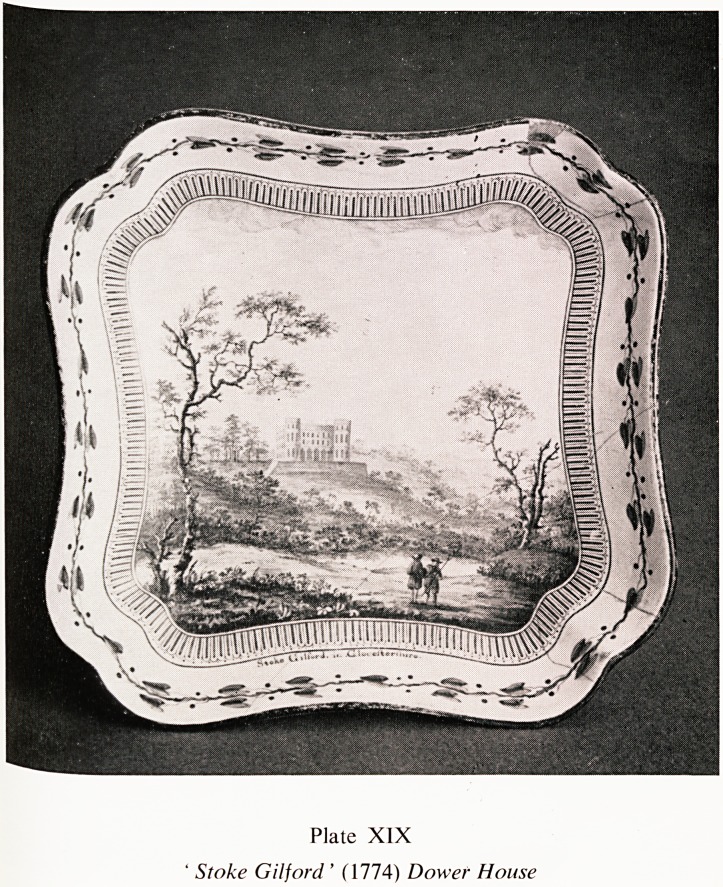


**Plate XX f7:**
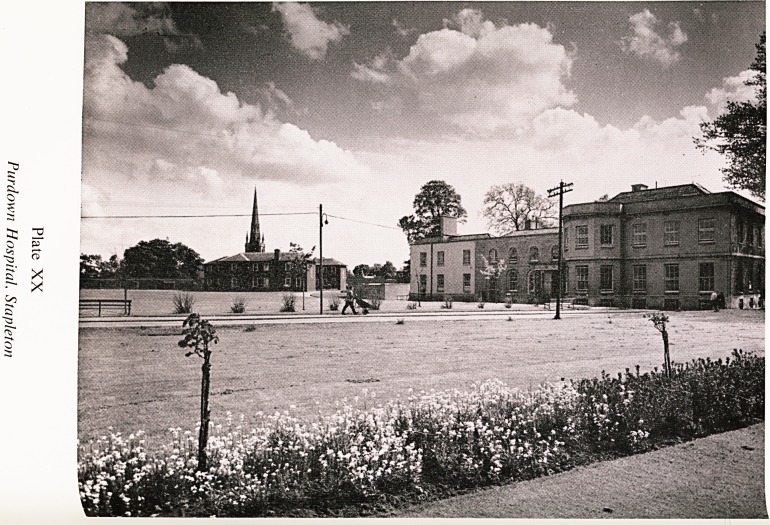


**Plate XXI f8:**
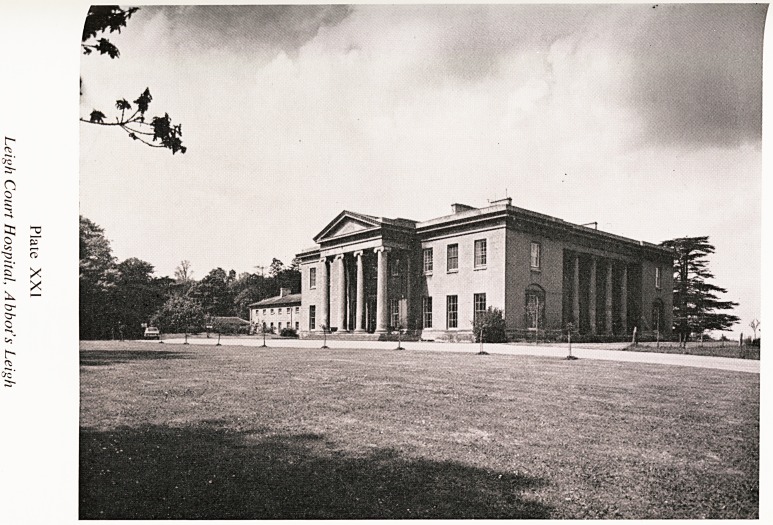


**Plate XXII f9:**